# French-language version of the World Health Organization quality of life spirituality, religiousness and personal beliefs instrument

**DOI:** 10.1186/1477-7525-10-39

**Published:** 2012-04-19

**Authors:** Olfa Mandhouj, Jean-François Etter, Delphine Courvoisier, Henri-Jean Aubin

**Affiliations:** 1 INSERM U699, Paris, France; 2 Association de santé mentale du 13ème arrondissement de Paris, Paris, France; 3 Institute of Social and Preventive Medicine, Faculty of Medicine, University of Geneva, 1 rue Michel-Servet, CH-1211, Geneve 4, Switzerland; 4 Division of Clinical Epidemiology, Geneva University Hospitals, Geneva, Switzerland; 5 Université Paris-Sud, Service d’addictologie, Hôpital Paul Brousse, Villejuif, France

**Keywords:** Spirituality, Religiousness, Quality of life, Internet surveys, Validity

## Abstract

**Background:**

A valid assessment of spirituality and religiousness is necessary for clinical and research purposes. We developed and assessed the validity of a French-language version of the World Health Organization Quality of Life Spirituality, Religiousness and Personal Beliefs Instrument (WHOQOL-SRPB).

**Methods:**

The SRPB was translated into French according to the methods recommended by the WHOQOL group. An Internet survey was conducted in 561 people in 2010, with follow-up 2 weeks later (n = 231, 41%), to assess reliability, factor structure, social desirability bias and construct validity of this scale. Tests were performed based on item-response theory.

**Results:**

A modal score of 1 (all answers=”not at all”) was observed for Faith (in 34% of participants), Connectedness (27%), and Spiritual Strength (14%). All scales had test-retest reliability coefficients ≥0.7. Cronbach’s alpha coefficients were high for all subscales (0.74 to 0.98) and very high (>0.9) for three subscales (Connectedness, Spiritual Strength and Faith). Scores of Faith, Connectedness, Spiritual Strength and Meaning of Life were higher for respondents with religious practice than for those who had no religious practice. No association was found between SRPB and age or sex. The Awe subscale had a low information function for all levels of the Awe latent trait and may benefit from inclusion of an additional item.

**Conclusions:**

The French language version of the SRPB retained many properties of the original version. However, the SRPB could be improved by trimming redundant items. The strength of SRPB relies on its multinational development and validation, allowing for cross-cultural comparisons.

## Background

The association between spirituality and health is an emerging area of research, relatively little explored in Europe [1-3]. Spirituality and religious involvement have been linked to positive health outcomes and to better quality of life [1,4-6]. In particular, spirituality and religiousness are associated with lower rates of physical, mental and substance use disorders and with how patients cope with illness [1,4-6]. Incorporating spiritual perspectives as a component of quality of life may allow for the implementation of better and possibly more acceptable health care, particularly for religiously oriented and/or terminally ill patients. Research on spirituality, religiousness and health has largely been conducted in North America [1], but religiousness and its relationship to health status varies greatly across countries [2,3]. Therefore, instruments that assess spirituality and religiousness ought to integrate input from various cultures and religions [6].

With this in mind, the World Health Organization Quality of Life Project developed the *Spirituality, Religiousness and Personal Beliefs Instrument* (WHOQOL-SRPB), a questionnaire that assesses quality-of-life aspects related to spirituality, religiousness and personal beliefs [6]. The SRPB is a chapter of the WHOQOL, which is a subjective, cross-cultural quality-of-life questionnaire and one of the few such instruments that includes an existential component. The SRPB was developed from an extensive research project conducted in 18 centers around the world, involving participants that represented all major religions [6]. However, the partial validation study of the SRPB (principal component analysis, internal consistency and some tests of construct validity) did not include any French-speaking country. France and Switzerland both have a secular culture and a high prevalence of agnosticism and atheism and, in this regard, they differ from many other countries [3]. Because there have been relatively few studies of associations between spirituality and health in these two countries, we set out to develop and assess the validity of a French-language version of the SRPB.

## Methods

### Study design

This study involved translating the WHOQOL-SRPB instrument from English into French, then assessing the validity of the French version by posting it on a French-language website and inviting participants to complete the questionnaire. Responses were analyzed to assess the reliability, factor structure, social desirability bias and construct validity of the French SRPB. The questionnaire is available at: http://www.stoptabac.ch/cgi-bin/spiritu.pl?language=fr. The study was approved by the ethics committee of the Pitié Hospital in Paris.

### The spirituality, religiousness and personal beliefs instrument

For research purposes, the construct of spirituality needs to be defined, operationalized and measured. The definition of spirituality has changed over recent decades, and its currently accepted meaning expands beyond religiosity. Differentiating spirituality from religiosity can be useful, particularly in secular countries where religiosity levels are low. Spirituality can be defined as experiences that seek to transcend self and to find meaning and purpose through connection with others, nature, and/or a supreme being; these experiences may or may not involve religious structures or traditions [7]. Spirituality, like personality and character, is an attribute of individuals. Religiosity, in contrast, refers to a link with an organized social entity [8]. In health care settings, spirituality has been studied in people from diverse religious backgrounds and in people with no religious background [9]. To avoid using a misleading dichotomous classification, spirituality is best understood as a multidimensional construct in which every individual can be located [10]. Like personality, culture or cognition, spirituality can be classified as a latent construct that cannot be observed directly but is inferred from observations of its component dimensions [11].

Taking these considerations into account, the SRPB was developed to evaluate how spirituality, religiosity and personal beliefs are related to quality of life in health and health care. The SRPB is a self-administered questionnaire that covers eight dimensions: Spiritual Connectedness, Meaning and Purpose in Life, Experience of Awe and Wonder, Wholeness and Integration, Spiritual Strength, Inner Peace, Hope and Optimism, and Faith. The SRPB includes 32 questions (four questions per dimension), answered on 5-point Likert-type scales that range from "not at all" to "an extreme amount" [6].

### Translation to French

The translation of the SRPB from English to French was performed according to the procedures recommended by the WHOQOL Working Group [12]. The first step was to perform a professional translation of the source instrument. Then, a bilingual panel reviewed the translation, looking for inconsistencies between the English and French versions. Next, a monolingual group assessed the French document, looking for aspects that were not clearly comprehensible or were ambiguous in French. This group commented on the style of questioning and discussed the instructions to respondents with a member of the bilingual panel. After incorporation of all corrections proposed by the monolingual group, the new French version was translated back into English. Comparison with the original English language version was judged to be satisfactory and did not lead to any corrections in the French version. The French questionnaire was then pre-tested in face-to-face interviews with 30 people, to check for comprehensibility and clarity, to improve the formulation of questions and to conduct a preliminary statistical analysis. After these pre-tests, a final version of the French SRPB was prepared for the current study (Table 1). Scores for the SRPB were computed as means of the items in each scale.

**Table 1:** French-language version of the WHOQOL-SRPB

**Questionnaire sur la spiritualité, la religion et les croyances personnelles**
**L’espoir et l’optimisme**
1. Est ce que vous vous sentez optimiste ?
2. Etes-vous optimiste en ce qui concerne votre vie ?
3. Votre optimisme améliore-t-il la qualité de votre vie ?
4. Etes-vous capable de rester optimiste dans les moments d'incertitude ?
**Le sens de la vie**
5. Percevez-vous un sens à la vie, d'une façon générale ?
6. Le fait de vous occuper des autres donne-t-il un sens à votre vie ?
7. Est-ce que vous ressentez que votre vie a un but?
8. Pensez-vous qu'il existe une raison pour que vous soyez présent sur terre ?
**L’émerveillement**
9. Etes-vous capable de vous émerveiller de ce qui vous entoure ? (Nature, art, musique)
10. Vous sentez-vous spirituellement touché par la beauté ?
11. Avez-vous des sentiments d'inspiration ou d'excitation dans votre vie ?
12. Avez-vous un sentiment de reconnaissance quand vous pensez aux choses qui vous font plaisir dans la nature ?
**La paix intérieure**
13. Vous sentez-vous en paix avec vous-même ?
14. Ressentez-vous une paix intérieure ?
15. Etes-vous capable de vous sentir paisible quand vous en avez besoin ?
16. Avez-vous l'impression qu'il existe une harmonie dans votre vie ?
**La plénitude**
17. Ressentez-vous une connexion entre votre esprit, votre corps et votre âme ?
18. Etes-vous satisfait de l'équilibre entre votre esprit, votre corps et votre âme ?
19. Avez-vous le sentiment que ce que vous vivez est en accord avec ce que vous pensez et ce que vous ressentez ?
20. Vos convictions personnelles vous aident-elles à créer une cohérence entre ce que vous faites, ce que vous pensez et ce que vous ressentez ?
**La puissance spirituelle**
21. A quel point ressentez-vous de la force spirituelle intérieure ?
22. Pouvez-vous trouver de la force spirituelle dans les moments difficiles ?
23. A quel point cette force spirituelle vous aide-t-elle à mieux vivre ?
24. Votre force spirituelle vous aide-t-elle à vous sentir heureux dans la vie ?
**La connexion à un être ou à une force spirituel(le)**
25. Le fait de vous connecter à un être spirituel (Dieu, une puissance supérieure, une énergie, ou autre) vous aide-t-il à traverser les moments difficiles ?
26. Le fait de vous connecter à un être spirituel vous aide-t-il à supporter le stress ?
27. Le fait de vous connecter à un être spirituel vous aide-t-il à comprendre les autres ?
28. Le fait de vous connecter à un être spirituel vous apporte-t-il du réconfort ?
**La foi**
29. Dans quelle mesure la foi contribue-t-elle à votre bien-être ?
30. Dans quelle mesure la foi vous apporte-t-elle du réconfort dans votre vie quotidienne ?
31. Dans quelle mesure la foi vous donne-t-elle de la force dans la vie quotidienne ?
32. Dans quelle mesure la foi vous aide-t-elle à apprécier la vie ?

*Réponses* : pas du tout = 1, un peu = 2, modérément = 3, beaucoup = 4, fortement = 5.

*Instructions* : Les questions suivantes portent sur vos croyances spirituelles, religieuses et personnelles et leur impact sur la qualité de votre vie. Les questions portent sur votre vécu des 2 dernières semaines. Veuillez répondre à toutes les questions. Si vous n'êtes pas sûr de votre réponse, mettez la réponse qui vous parait la plus appropriée.

### Validity assessment

We conducted an Internet survey to assess the validity of the translated instrument, with a follow-up (retest) two weeks later. The survey form, in French, was posted on the smoking cessation website Stop-Tabac.ch [13,14] for five months (from December 2009 to May 2010). This site was chosen for convenience. Participants were informed that their answers would be stored on a computer file for statistical analyses, and were given the option to request that their answers were not retained on file. Participants who wanted to take part in a follow-up survey two weeks later indicated their e-mail address.

The psychometric characteristics of the French-language version of the SRPB were examined by studying the response distributions (missing values, normality, floor and ceiling effects, central tendency), and by assessing reliability and factor structure. Respondents also indicated whether they were sure of their answers to each question (not at all, moderately, absolutely sure). We conducted tests of construct validity (associations with religiosity and with life-threatening health problems), and we used the item response theory (IRT) to examine how precise and informative the scale and subscales were for respondents with different levels of spirituality.

Item response theory analyses estimate two types of parameters. The first type is called *person parameter* which indicates each participant’s latent score on the underlying construct (in this case, spirituality). The person parameter is estimated on a standardized scale, with extremely low spirituality represented by -4 and extremely high spirituality represented by +4, and indicates the probability of scoring highly on each item. The second type of parameter is concerned with the items (*item parameters*), e.g., item severity indicates the level of “difficulty” of each item. An item with a negative item parameter has a high probability of being answered with a high score, whereas an item with a positive item parameter will probably yield a low score [15]. Based on theory, we assumed that the total SRPB scale and each subscale would measure a single dimension. This assumption is commonly made when the mean value or the sum of the items is used to compute a score. Based on the person parameters and item parameters, we obtained total information curves, which depict the information value of each scale at each level of the person parameters; higher information values denote more precision. The lower boundary of the information function is zero, which indicates that the scale provides no information on the underlying construct at a given level of person parameter. There is no upper boundary, as this value depends on the number of items and modalities.

### Reliability

To assess test-retest reliability, intra-class correlation coefficients were computed for each item and subscale, using the two measures (baseline and two weeks later). We also checked whether the internal consistency coefficients (Cronbach's alpha) of the SRPB general scale and subscales exceeded 0.7, as recommended [16]. For each item, the alpha coefficients of the corresponding subscale were assessed if this item had been deleted.

### Factor analysis

To assess the structure of SRPB, exploratory factor analysis with promax rotation was applied. To determine the number of factors to retain, Velicer's MAP test, Horn's parallel analysis, the criterion of eigenvalue >1, and criteria of interpretability were applied [17].

### Social desirability bias

To assess social desirability bias (the tendency to give answers that conform to a perceived social norm), correlations between SRPB ratings and the Marlowe-Crowne Social Desirability scale (13-item short form, French-language version) were assessed [18,19].

### Tests of construct validity

Associations between SRPB scores and either religious affiliation or religious practice were assessed, as we expected to observe higher SRPB scores in participants who indicated a religious affiliation (Catholic, Protestant, Jewish, Muslim or Buddhist) than in atheists, and higher scores in participants who had a regular religious activity than in those who had none and had no feeling of religious belonging. We also compared SRPB scores in people who ever had a life-threatening health problem (n = 103) with those who never had such a problem (n = 410). Any association between SRPB ratings and sex, age and education (possession of a diploma that would give access to university, or not) [6] were also assessed.

### Statistical analyses

The following statistical tests were used: *t* tests to compare means in two samples, *F* tests from analysis of variance to compare means in more than two samples, and chi-square tests to compare proportions. Linear regression models were used to assess associations between SRPB scores and age. Explanatory factor analysis and IRT analyses were conducted using R statistical software, and all other analyses were conducted with SPSS.

## Results

### Participant characteristics

There were 561 participants at baseline and 231 (41%) at the 2-week follow-up. Most respondents were female, most (71%) had an educational diploma that would give access to university, most (76%) had no religious practice, and 37% were either agnostics or atheists. Participants lived in France (57%), Switzerland (24%), Belgium (7%), Canada (4%) and other countries (8%). The majority of participants were former smokers and there were few never smokers (Table 2).

**Table 2:** Characteristics of study participants who responded to French-language SRPB, Internet 2009-2010

**Characteristic**	
Number of respondents	561
Age, years (mean, SD)	42.2 (11.6)
Men (%)	33.9
Obtained a diploma giving access to university (%)	70.6
Professional status (%)	
Professional	29.9
Employee	29.9
Intermediary profession	12.3
No professional activity	10.6
Retired	5.7
Craftsman	5.3
Other, non response	6.3
How would you qualify your religious practice? (%)	
Regular	11.6
Occasional	10.7
None, but feeling of belonging	33.2
None, and no feeling of belonging	42.8
To which religious affiliation do you feel closest? (%)	
Catholic	28.7
Protestant	8.0
Jewish	2.0
Islam	4.1
Buddhist	14.8
Agnostic	18.7
Atheist	18.4
Did you ever have a serious health problem? (Yes, %)	25.3
If you did, did you ever think that your life was threatened by this health problem?	72.5
Smoking status (%)	
Daily smoker	28.3
Occasional (non-daily) smoker	3.9
Former smoker	58.3
Never smoker	7.3

### Missing values

No single SRPB item produced more than 4.3% of missing values. The eight subscales could be computed for 98.8-100% of respondents. The general scale could be computed for all respondents.

### Floor and ceiling effects

A modal score of 1 (all answers=“not at all”) was observed for Faith (in 34.2% of respondents), Connectedness (26.6%), and Spiritual Strength (13.5%). The distributions of the Awe and Hope subscales were slightly skewed to the right (skewness: Awe = -0.56, Hope = -0.54), but nevertheless did not present a substantial ceiling effect. Faith was skewed to the left (skewness = 0.41) because of a modal “not at all” answer. The other subscales and the general SRPB score had roughly symmetrical distributions (graphs not shown).

### Test-retest reliability

With the exception of the Awe scale (test-retest correlation = 0.69), all scales had test-retest reliability coefficients above 0.7. However, a few items, in particular from the Hope subscale, had relatively low test-retest coefficients (r < 0.6) (Table 3).

**Table 3:** Psychometric properties of the French-language version of the WHOQOL-SRPB

***Abbreviated item content***	**Correlation with social desirability**	**Test-retest reliability coefficient**	**Cronbach’s alpha or alpha if item deleted**^**a**^	**Rather sure + not sure of their answer (%)**	**“Not at all”** ** (%)**	**“An extreme amount” (%)**	**Mean (SD)**
**Hope & Optimism**							
1. Hopeful	0.23***	0.59***	0.72	39.0	5.2	8.6	3.25 (0.94)
2. Hopeful about life	0.25***	0.73***	0.72	35.1	3.9	8.9	3.29 (0.94)
3. Optimistic, quality of life	0.15***	0.48***	0.80	24.4	4.6	24.1	3.67 (1.05)
4. Optimistic in uncertainty	0.28***	0.63***	0.77	32.9	11.4	3.4	2.80 (0.91)
**Meaning of life**							
5. Finds meaning in life	0.22***	0.72***	0.69	27.7	10.7	20.0	3.39 (1.22)
6. Taking care of others	0.21***	0.58***	0.82	20.6	3.7	25.5	3.75 (0.94)
7. Life has a purpose	0.19***	0.76***	0.66	29.0	15.3	20.3	3.16 (1.29)
8. Here for a reason	0.12**	0.74***	0.77	29.9	28.3	21.7	2.95 (1.46)
**Awe**							
9. Experiences awe	0.18***	0.61***	0.70	11.9	0.7	50.4	4.23 (0.81)
10. Touched by beauty	0.08 ns	0.69***	0.68	22.6	8.2	30.8	3.70 (1.14)
11. Feelings of inspiration	0.06 ns	0.60***	0.66	24.9	2.5	21.9	3.59 (0.89)
12. Grateful for things	0.17 ***	0.73***	0.69	24.0	10.2	23.9	3.54 (1.23)
**Inner Peace**							
13. Peaceful with yourself	0.27***	0.78***	0.85	31.9	9.4	11.1	3.19 (1.07)
14. Has inner peace	0.25***	0.73***	0.83	31.4	14.4	8.6	2.98 (1.11)
15. Feels peaceful	0.29***	0.66***	0.87	33.7	9.2	6.0	2.94 (0.94)
16. Sense of harmony	0.23***	0.73***	0.87	34.4	11.6	8.0	3.07 (0.96)
**Wholeness**							
17. Connection mind body	0.21***	0.76***	0.81	30.9	20.7	16.8	3.10 (1.27)
18. Balance mind body soul	0.22***	0.66***	0.73	33.4	20.0	7.1	2.77 (1.18)
19. Way you live consistent	0.28***	0.61***	0.75	29.7	14.8	8.9	3.07 (1.10)
20. Creates coherence	0.19***	0.54***	0.77	27.4	5.9	18.4	3.36 (1.07)
**Spiritual Strength**							
21. Feels spiritual strength	0.19***	0.75***	0.94	31.9	19.6	12.8	2.96 (1.30)
22. Strength difficult times	0.18***	0.81***	0.92	28.7	18.4	10.3	2.88 (1.24)
23. Helps to live better	0.11***	0.86***	0.91	27.6	19.4	12.7	2.98 (1.34)
24. Feels happy in life	0.14***	0.76***	0.92	27.9	22.1	10.5	2.86 (1.32)
**Connectedness**							
25. Gets through hard times	0.12**	0.82***	0.93	22.3	32.8	16.0	2.71 (1.49)
26. Tolerates stress	0.14***	0.80***	0.93	26.3	43.5	6.2	2.33 (1.30)
27. Understands others	0.16***	0.83***	0.93	25.2	42.1	9.8	2.48 (1.42)
28. Comforts, reassures	0.12**	0.87***	0.92	24.4	36.9	12.5	2.56 (1.47)
**Faith**							
29. Contributes well-being	0.15***	0.92***	0.97	23.0	38.9	10.7	2.51 (1.46)
30. Gives you comfort	0.18***	0.91***	0.97	22.6	39.9	7.7	2.37 (1.40)
31. Gives you strength	0.17***	0.89***	0.97	20.3	39.8	9.8	2.42 (1.43)
32. Helps you enjoy life	0.17***	0.88***	0.98	20.0	39.8	12.1	2.49 (1.48)
					**Skew**	**Kurtosis**	
Hope & Optimism	0.29***	0.75**	0.80	--	-0.54	0.24	3.33 (0.78)
Meaning of Life	0.21***	0.79***	0.79	--	-0.24	-0.79	3.33 (1.04)
Awe	0.15***	0.69***	0.74	--	-0.56	0	3.84 (0.81)
Inner Peace	0.30***	0.81***	0.89	--	-0.20	-0.56	3.02 (0.95)
Wholeness	0.27***	0.76***	0.81	--	-0.15	-0.70	3.07 (0.97)
Spiritual Strength	0.15***	0.84***	0.94	--	-0.22	-1.11	2.94 (1.22)
Connectedness	0.15***	0.88***	0.95	--	0.29	-1.37	2.51 (1.35)
Faith	0.17***	0.92***	0.98	--	0.41	-1.29	2.43 (1.39)
**Total score, 32 items**	0.22***	0.90***	0.96	--	0.09	-0.78	3.05 (0.83)

^a^. Cronbach’s alpha for scales. For each item, alpha of the corresponding subscale if this item was deleted.

* *p* ≤ 0.05; ** *p* ≤ 0.01; *** *p* ≤ 0.001.

### Internal consistency

Cronbach’s alpha coefficients were high for all subscales (range 0.74 to 0.98), and very high (>0.9) for three subscales (Connectedness, Spiritual Strength and Faith) (Table 3).

### Factor analyses

The rule of eigenvalue > 1 suggested retention of all eight factors, Horn’s parallel analysis three factors and Velicer’s MAP test seven factors. An eight–factor solution was interpretable and explained 65% of the variance (Table 4). All the SRPB *a priori* dimensions loaded on distinct factors and were well defined, except for the Wholeness dimension, which loaded on two different factors, and for the Connectedness dimension, which loaded higher on the first factor than on its specific factor. Moreover, the item “taking care of other people” did not load on the expected factor, leading the Meaning of Life dimension to be defined by only three items. There were substantial correlations between Faith and Connectedness (r = 0.88); Faith and Spiritual Strength (r = 0.73); Connectedness and Spiritual Strength (r = 0.77); and Inner Peace and Wholeness (r = 0.74) (*p* < 0.001 for all these correlations).

**Table 4:** Factor structure of the French-language version of the WHOQOL-SRPB

***Abbreviated item content***	**Factor 1**	**Factor 2**	**Factor 3**	**Factor 4**	**Factor 5**	**Factor 6**	**Factor 7**	**Factor 8**
**Hope & Optimism**								
1. Hopeful				85				
2. Hopeful about life				74				
3. Optimistic, quality of life				62				35
4. Optimistic in uncertainty		32		65				
**Meaning of life**								
5. Finds meaning in life						83		
6. Taking care of others								88
7. Life has a purpose						83		
8. Here for a reason						69		
**Awe**								
9. Experiences awe					90			
10. Touched by beauty			47		59			
11. Feelings of inspiration					77			
12. Grateful for things					40			
**Inner Peace**								
13. Peaceful with yourself		96						
14. Has inner peace		82						
15. Feels peaceful		92						
16. Sense of harmony		49						
**Wholeness**								
17. Connection mind body			49					
18. Balance mind body soul		59					31	
19. Way you live consistent							73	
20. Creates coherence							79	
**Spiritual Strength**								
21. Feels spiritual strength			79					
22. Strength difficult times			83					
23. Helps to live better			87					
24. Feels happy in life			77					
**Connectedness**								
25. Gets through hard times	69							
26. Tolerates stress	78							
27. Understands others	82							
28. Comforts, reassures	86							
**Faith**								
29. Contributes well-being	100							
30. Gives you comfort	100							
31. Gives you strength	99							
32. Helps you enjoy life	96							

Factor loadings x 100, loadings below 30 are not shown.

### Social desirability

Correlations between the social desirability score and all subscales were significant at the *p* = 0.001 level, but these correlations were relatively small (all r≤0.30). The largest correlations were observed for Inner Peace (r = 0.30) and Hope (r = 0.29).

### Confidence

Substantial proportions (>20%) of “not sure” and “rather sure” answers were observed for items in all subscales (Table 3).

### Tests of construct validity

The 65 respondents who had a regular religious practice were compared with the 240 who reported having no religious practice and no feeling of belonging. Scores of Faith, Connectedness, Spiritual Strength and Meaning of Life were significantly higher for respondents with religious practice. Smaller differences were observed for Hope, Awe and Inner Peace (Table 5).

**Table 5:** Tests of construct validity for the French-language version of the WHOQOL-SRPB

***Abbreviated item content***	**Differences between regular vs no religious practice or belonging**	**Differences between religious affiliation vs. atheists**	**Ever vs. never had a life-threatening health problem**	**Diploma giving access to University(difference between yes/no)**
**Hope & Optimism**				
1. Hopeful	0.34**	0.17 ns	-0.09 ns	-0.18*
2. Hopeful about life	0.46**	0.16 ns	-0.06 ns	-0.12 ns
3. Optimistic, quality of life	0.51**	0.32**	0.20 ns	-0.18 ns
4. Optimistic in uncertainty	0.30*	0.16 ns	-0.07 ns	-0.06 ns
**Meaning of life**				
5. Finds meaning in life	1.17***	0.66 ***	0.13 ns	-0.14
6. Taking care of others	0.53**	0.37**	0.36 **	-0.07
7. Life has a purpose	1.36***	0.74***	0.27 ns	-0.21
8. Here for a reason	2.05***	1.54***	0.17 ns	-0.50***
**Awe**				
9. Experiences awe	0.18 ns	0.17*	0.19 *	0.07
10. Touched by beauty	0.85***	0.82***	0.22 ns	0.04
11. Feelings of inspiration	0.30*	0.30**	0.15 ns	-0.11
12. Grateful for things	0.96**	1.02***	0.39 **	-0.35**
**Inner Peace**				
13. Peaceful with yourself	0.59***	0.09 ns	-0.06 ns	-0.03
14. Has inner peace	1.07***	0.35**	-0.15 ns	-0.01
15. Feels peaceful	0.63***	0.23*	0.07 ns	-0.07
16. Sense of harmony	0.71***	0.30*	-0.20 ns	-0.12
**Wholeness**				
17. Connection mind body	1.32***	1.07***	0.18 ns	-0.24
18. Balance mind body soul	0.94***	0.52***	-0.17 ns	-.012
19. Way you live is consistent	0.62***	0.17 ns	-0.01 ns	0.02
20. Creates coherence	0.69***	0.25*	-0.05 ns	0.05
**Spiritual Strength**				
21. Feels spiritual strength	1.67***	1.28***	0.26 ns	-0.20
22. Strength in difficult times	1.88***	1.30***	0.20 ns	-0.14
23. Helps live better	1.96***	1.46***	0.23 ns	-0.19
24. Feels happy in life	1.84***	1.38***	0.07 ns	-0.21
**Connectedness**				
25. Gets through hard times	2.61***	2.03***	0.22 ns	-0.39**
26. Tolerates stress	2.04***	1.57***	0.10 ns	-0.37**
27. Understands others	2.59***	1.73***	0.11 ns	-0.32**
28. Comforts, reassures	2.62***	1.96***	0.12 ns	-0.48***
**Faith**				
29. Contributes to well-being	2.87***	1.94***	0 ns	-0.35**
30. Gives you comfort	2.67***	1.76***	0.01 ns	-0.46***
31. Gives you strength	2.64***	1.85***	0.07 ns	-0.38**
32. Helps you enjoy life	2.69***	1.93***	0.02 ns	-0.48***
Hope & Optimism	0.40***	0.20*	0 ns	-0.14 ns
Meaning of Life	1.30***	0.84***	0.24 *	-0.22*
Awe	0.58***	0.58***	0.25 **	-0.08 ns
Inner Peace	0.76***	0.25*	-0.08 ns	-0.05 ns
Wholeness	0.89***	0.47***	-0.01 ns	-0.08 ns
Spiritual Strength	1.83***	1.35***	0.21 ns	-0.18 ns
Connectedness	2.45***	1.83***	0.14 ns	-0.40**
Faith	2.71***	1.87***	0.02 ns	-0.40**
**Total score, 32 items**	1.35***	0.91***	0.09 ns	-0.19*

* *p* ≤ 0.05; ** *p* ≤ 0.01; *** *p* ≤ 0.001.

In addition, the 323 respondents who declared a religious affiliation (Catholic, Protestant, Jewish, Muslim or Buddhist) were compared with the 103 who declared themselves to be atheists. Large differences between these two groups were observed for Faith and Connectedness, and smaller differences for Hope, Inner Peace and Wholeness (Table 5).

People who ever had a life-threatening health problem had slightly elevated scores for Awe and Meaning of Life, compared with those who never had such a problem. In contrast with previous reports [6], we found no association between SRPB scores and either age or sex (data not shown). However, negative associations were found between education (possession of a diploma giving access to university, or not) and the Connectedness and Faith scales, and some items in the Meaning of Life and Awe scales (Table 5).

### Item response theory analysis

**Figure 1:**
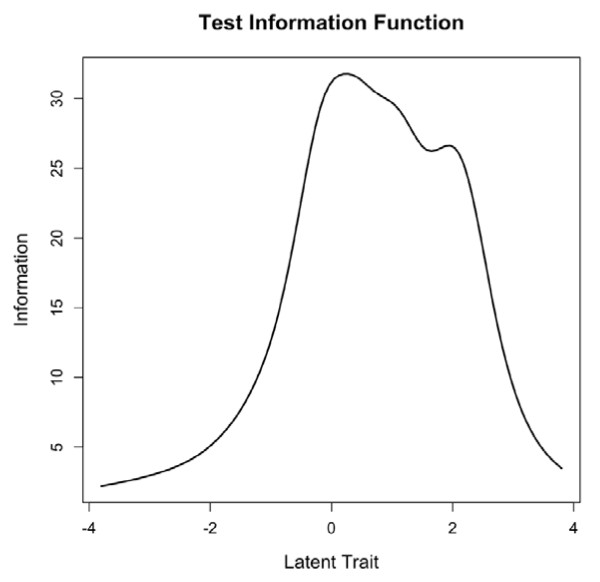
Test information function for the latent spirituality trait (total score).

**Figure 2:**
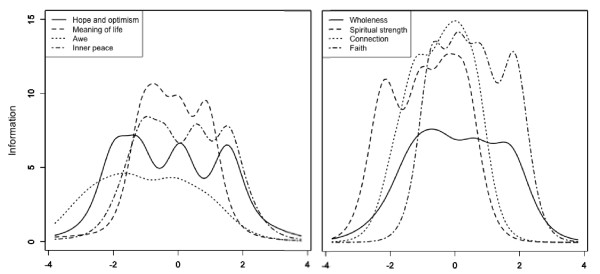
Test information function for the SRPB subscales.

Item response theory analysis showed that only 79 respondents (14%) had low spirituality scores (-1 or below), 431 (77%) had slightly low to high spirituality scores (between -1 and +2), and 51 (9%) had very high spirituality scores (+2 and above). The information function (Figure 1) showed that the total score (i.e., all items) was most informative for respondents in the middle range of values (from -1 to +2). As all the subscales have the same number of items and response options, the maximum value of their information function is the same and curves can be compared. The information function of the subscales (Figure 2) showed that the Awe subscale, and to a lesser degree, the Hope, Inner Peace and Wholeness subscales were not very informative. This indicates that an individual’s answers to any question can be almost perfectly predicted from his/her answers to the other questions on the scale, and that the additional questions are thus redundant and not informative.

## Discussion

We developed a French-language version of the WHOQOL-SRPB and assessed its validity. The translated version retained many of the properties of the original version. In particular, the French version produced few missing answers, its test-retest reliability coefficients and alpha coefficients were high, and its factor structure was interpretable. Some limitations of this translated scale were also apparent. For example, the original factor structure was not present in our data, there may be some redundancy among items, and the scale could probably be trimmed without losing much information. With 32 questions, the SRPB is rather long, and a shorter version could be useful for studies in which spirituality is only one of several measures.

### Number of factors

The authors of the original English-language SRPB scale did not indicate what rule they had used to identify the number of factors in the scale, nor did they report results of a confirmatory factor analysis [6]. Our analyses suggest that the scale may contain fewer than eight factors, as Faith, Connectedness and Spiritual Strength were highly correlated, as were Inner Peace and Wholeness. Models with fewer dimensions and items may need to be explored.

### Reliability

Cronbach’s alpha coefficients for the subscales were high (0.74 to 0.98) for the French version and comparable to alpha coefficients reported in the original publication (0.77 to 0.95) [6]. However, for several subscales, alpha coefficients were perhaps too high (>0.9), which suggests that there is some redundancy and that several items could probably be deleted. Items that had low test-retest coefficients, and those for which participants were not sure of their answers might be either rewritten or deleted.

### Social desirability

Correlations with social desirability ratings were somewhat higher than expected, which suggests that at least some of the variance in the SRPB can be explained by social desirability, particularly for the Inner Peace and Hope scales.

### Construct validity

As expected, scores for Faith and Connectedness were substantially higher for religious participants than for either atheists or participants who reported having no religious practice or sense of belonging. However, relatively small differences were observed between these groups for Hope and for Inner Peace, and differences for several items in these scales were non-significant. These findings support the concept that spirituality can stretch outside religiosity, leaving the possibility for a category of individuals who have high spirituality ratings even though they are not religious [9].

Having ever had a life-threatening health problem was associated with elevated scores on some items only (in particular, feeling grateful and taking care of others). No association was found between gender and SRPB scores in our study, even though several studies have found that women are generally more religious than men [20,21]. However, the associations previously reported between gender and SRPB were quite weak (about 0.1 standard deviation units) [6], and thus, our data do not necessarily contradict these earlier findings.

### Information function

Item response theory analyses showed that the total score was most informative for individuals with medium to high spirituality scores, but was less informative for those with very low spirituality scores, which is consistent with the fact that most of the participants acknowledged a religious affiliation. The scale would not be able to discriminate well between individuals with slightly low (e.g., -1) and very low (e.g., -3) spirituality ratings. While most subscales were at least adequately informative, the Awe subscale had a low information function for all levels of the Awe latent trait and may benefit either from inclusion of an additional item, or from rewriting.

### Study limitations

This study was conducted in a self-selected sample of individuals who were users of a smoking cessation website. This method over-sampled current and former smokers, women and people with higher education, under-sampled never smokers [13,22], and included mostly healthy people. Thus, although our study provides useful information on the performance of the SRPB, our findings should be interpreted with caution, because they may not be generalizable to other populations in which the SRPB is likely to be used (e.g. very sick people, the elderly, or people without a higher education). It is not clear whether and how the inclusion of a majority of ever smokers affected our results, but some research suggests that religiousness and spirituality may protect against smoking [23,24]. Testing this scale in representative samples, in illness samples, in different countries and cultural groups, is warranted.

## Conclusion

Relatively few scales specifically measure spirituality and religiousness [6]. The strength of the SRPB relies on its multinational and multi-language development and validation, which allows cross-cultural comparisons. The French language version of the SRPB retained many characteristics of the original, English-language version, and was found to meet tests of reliability and construct validity. However, the SRPB could be improved by trimming redundant items or rewriting some items.

## Abbreviations

WHOQOL, World Health Organization quality of life; SRPB, spirituality, religiousness and personal beliefs instrument; IRT, item response theory; MAP, minimum average partial.

## Competing interests

The authors declare that they have no competing interests

## Authors' contributions

OM, HJA and JFE conceived of the study and designed it. JFE and DC performed the statistical analysis. JFE was in charge of data collection. All authors contributed to writing the manuscript. All authors read and approved the final manuscript.
